# Mild-to-moderate depressive symptoms impact on self-reported outcome measures in clinical trials for neurodegenerative diseases

**DOI:** 10.1177/17407745251387571

**Published:** 2025-11-30

**Authors:** Christine Girges, Nirosen Vijiaratnam, Alexa King, Grace Auld, Rachel McComish, Kashfia Chowdhury, Gareth Ambler, Kate Maclagan, Patricia Limousin, Dilan Athauda, Huw R Morris, Vincenzo Libri, Thomas Foltynie, The Exenatide PD3 and MSA Trials Consortia

**Affiliations:** 1 Department of Clinical and Movement Neurosciences, Institute of Neurology, University College London, London, UK; 2 Clinical Research Centre for Movement Disorders and Gait, Kingston Centre, Parkinson’s Foundation Centre of Excellence, Monash Health, Cheltenham, VIC, Australia; 3 Department of Medicine, School of Clinical Sciences, Monash University, Clayton, VIC, Australia; 4 The Comprehensive Clinical Trials Unit, University College London, London, UK; 5 Department of Statistical Science, University College London, London, UK; 6 The Francis Crick Institute, London, UK; 7 NIHR Clinical Research Facility, University College London Hospitals NHS Foundation Trust, London, UK

**Keywords:** Depressive symptoms, neurodegenerative diseases, outcome measures, clinical trials

## Abstract

**Introduction:**

Depression can be an intrinsic part of neurodegeneration, or a reaction to the onset of motor or non-motor disability. Depression can also adversely influence an individual’s perception of their disease, independently of its severity or impact on function. Participant-reported outcome measures are recognised as an important adjunct to objective clinical trial data. Although severe depression is a frequent contraindication for trial participation, mild-to-moderate depressive symptoms could potentially influence the outcome of such questionnaires. We aimed to explore this within two interventional trials for Parkinson’s disease (Exenatide PD3; NCT04232969) and multiple system atrophy (Exenatide MSA; NCT04431713).

**Methods:**

Prior to investigational drug exposure, participants completed either the Patient Health Questionnaire or Beck Depression Inventory-II, which allowed us to dichotomise them into two groups (normal or mild-to-moderately elevated burden of depressive symptoms). Participants with Parkinson’s self-completed the Movement Disorder Society Sponsored Revision of the Unified Parkinson’s disease Rating Scale Parts 1b and 2, Parkinson’s Disease Questionnaire-39, Non-motor Symptoms Scale and EQ-5D-5L, while participants with multiple system atrophy self-completed a Quality of Life scale and had assistance with completing the Unified Multiple System Atrophy Rating Scale Part 1. The Movement Disorder Society Sponsored Revision of the Unified Parkinson’s Disease Rating Scale Part 3 and Unified Multiple System Atrophy Rating Scale Part 2 provided objective, clinician-rated measures of motor severity.

**Results:**

A mild-to-moderately elevated burden of depressive symptoms was identified in 32.5% (63/194) and 42.0% (21/50) of Parkinson’s and multiple system atrophy participants, respectively. Despite the normal and elevated groups being comparable in terms of disease duration and objective motor severity, those with a mild-to-moderately elevated burden of depressive symptoms self-reported worse disease impact. However, measures which involved clinician and/or carer input (Unified Multiple System Atrophy Rating Scale Part 1) were not influenced by co-existing depressive symptoms.

**Conclusions:**

Mild-to-moderate depressive symptoms, below the threshold for diagnosing a depressive disorder, are associated with negative self-reporting, and such findings should be carefully considered when planning the design and analysis of trials in neurodegenerative diseases.

## Introduction

The prevalence of neurodegenerative diseases is rising due to the aging population globally, highlighting the unmet need for effective therapeutic interventions which relieve symptoms or halt disease progression. As such, there are many clinical trials investigating the efficacy and tolerability of both new and repurposed medicines as either symptomatic or disease-modifying treatments.^1–3^

These trials often exclude participants with concurrent severe depression to minimise (1) the risk of adverse events directly or indirectly related to mood and (2) potential disruption to data collection caused by missed appointments or high attrition rates. Depression may be an important non-motor symptom of a neurodegenerative process and should be appropriately captured to fully record the impact of an intervention on an individual’s clinical state. Beyond this, severe depression is also known to negatively influence the participant’s own perception and experience of their disease, perhaps independent of disease severity or physical function.^4–6^ It is unclear therefore the extent to which mild-to-moderate depressive symptoms could introduce a potential bias or otherwise influence the completion of self-reported outcome measures in trials, which are acknowledged as an important adjunct to objective measurements of disease severity.

We explored this in a cohort of participants with Parkinson’s disease and multiple system atrophy who were recruited into two disease-modifying trials of exenatide, a glucagon-like peptide-1 receptor agonist licenced to treat type 2 diabetes mellitus. Although concurrent severe depression formed an exclusion criterion for recruitment, it was anticipated that a proportion of included participants would still report mild-to-moderate depressive symptoms. Psychiatric comorbidities are common in both disease groups, often with a complex multifaceted aetiology. Depression can be a reactive psychological response to severe disability and a progressive loss of independence or social status, as well as part of the neurodegenerative process itself. The underlying pathophysiology likely encompasses neurotransmitter and/or trophic support dysregulation within limbic circuitry, neuroinflammation and/or a genetic predisposition.^7–11^

The aim of this study was not to disentangle the contributory causes of minor depressive symptoms, but to understand the extent to which their existence influences self-reported disease severity in comparison with objective ratings performed by trial doctors. Within this, we had to carefully consider whether the self-reported measures were in fact independently completed or if the presence of an additional person (e.g. caregiver) affected the scores. For this work, only data collected prior to investigational drug exposure were analysed.

## Methods

### Ethics statement

The London Hampstead Research Ethics Committee (20/LO/0473) and South-Central Berkshire Research Ethics Committee (19/SC/0447) granted ethical approval for the Exenatide MSA and Exenatide PD3 trials, respectively.

### Participants

The study eligibility criteria and trial design has been previously described.^12,13^ To summarise, participants with either a diagnosis of Parkinson’s or multiple system atrophy (according to the Gilman et al.^14^ criteria) were recruited into two randomised controlled trials of extended-release exenatide 2 mg between 2020 and 2022 in the United Kingdom (ClinicalTrial.gov: NCT04232969 and NCT04431713). Individuals with dementia or severe depression at screening (Patient Health Questionnaire (PHQ-9) ≥ 16 or Beck Depression Inventory-II (BDI-II) ≥ 30) were excluded from taking part, as were those with diabetes mellitus or a history of pancreatitis and thyroid cancers. Participants with multiple system atrophy were required to still be ambulant (with or without assistance) and were less than 5 years from the time of a documented diagnosis. Eligible participants were given a detailed description of the trial, and their written informed consent was obtained.

### Procedures

#### Parkinson’s disease

The following questionnaires were completed at the baseline visit: (1) The PHQ-9, a brief scale based on *Diagnostic and Statistical Manual of Mental Disorders* (Fourth edition; *DSM*-IV diagnostic criteria for depression. A score of 0–4 indicated minimal, 5–9 indicated mild, 10–14 indicated moderate, 15–19 indicated moderately severe and 20–27 indicated severe depression; (2) The Parkinson’s Disease Questionnaire-39 (PDQ-39), measuring health-related quality of life across eight dimensions. Each dimension total score ranged from 0 to 100, and an overall summary index could be computed by taking a weighted average of these scores; (3) the Non-motor Symptoms Scale (NMSS), measuring the severity and frequency of non-motor symptoms; and (4) The EQ-5D-5L, a generic health-status instrument comprising items on mobility, self-scare, usual activities, pain/discomfort and anxiety/depression, from which a summary index was calculated. A visual analogue scale recorded self-rated health, where 0 indicated the worst and 100 indicated the best health status. Higher scores on these scales represented more severe symptoms. The PDQ-39, NMSS and EQ-5D-5L contained mood related items.

The Movement Disorder Society Sponsored Revision of the Unified Parkinson’s Disease Rating Scale (MDS-UPDRS) assessed overall clinical severity. The motor examination (Part 3) was performed OFF and ON dopaminergic medication. Surrogate total scores were calculated using a validated imputation strategy for participants who had incomplete assessments (n = 2).^15^ Part 1b (patient-reported non-motor aspects of daily living), Part 2 (patient-reported motor aspects of daily living) and Part 4 (motor complications) were completed. As MDS-UPDRS Part 1a assessed neuropsychiatric symptoms (and would correlate highly with the PHQ-9), it was not analysed.

The MDS-UPDRS Parts 1b and 2 could be completed by the participant or with input from their caregiver. To avoid a potential bias, we excluded data that were derived from both participant and caregiver (n = 9). The PDQ-39 and EQ-5D-5L were entirely self-completed by the participant, while the NMSS and MDS-UPDRS Part 4 required a discussion between participant and clinician.

#### Multiple system atrophy

The following questionnaires at baseline were completed: (1) the BDI-II measured the severity of depression symptomatology. A score of 13 or less indicated minimal, 14–19 indicated mild, 20–28 indicated moderate and 29–63 indicated severe depression; and (2) the Multiple System Atrophy Quality of Life (MSA-QoL) scale measured health-related quality of life across three subscales (motor, non-motor and emotional/social functioning), with higher scores indicating a poorer quality of life This instrument included a visual analogue scale for overall life satisfaction, where 0 indicated extreme dissatisfaction and 100 indicated extreme satisfaction.^16^ Although the MSA-QoL can be completed by either the participant or their caregiver, all of our participants completed the MSA-QoL independently.

Overall clinical severity was assessed using the Unified Multiple System Atrophy Rating Scale (UMSARS). The first sub-score (UMSARS Part 1) captured participant-reported functional disability, which involved a discussion between participant, carer, and clinician to agree on the historical severity of the illness. The second sub-score (UMSARS Part 2) measured clinician-assessed motor impairment.

### Statistical analysis

Normality of the data was assessed using the Shapiro–Wilk test. Participants with Parkinson’s were dichotomised into two groups based on their baseline PHQ-9 score. A normal or elevated burden of depressive symptoms were defined as a PHQ-9 score of 0–4 or ≥5, respectively.^17^ Participants with multiple system atrophy were dichotomised into two groups based on their baseline BDI-II score. A normal or elevated burden of depressive symptoms was defined as a BDI-II score of 0–13 or ≥14, respectively.^18^

An independent samples t-test (for normally distributed continuous variables) or Mann–Whitney U test (for non-normally distributed continuous variables) was applied between the normal and elevated depressive symptom groups within each disease. Chi-square tests were used for categorical data. To further evaluate the impact of mild-to-moderate depressive symptoms on self-reported outcome measures of disease severity, we performed univariable linear regression analyses (including the PHQ-9 and BDI-II scores as continuous variables) and then constructed multivariable linear regression models, adjusting for confounders (age, gender with ‘male’ as the reference category, disease duration, objective motor severity, anti-depressant use with ‘no use’ as the reference category). This was only performed for questionnaires which were previously shown to be significantly different between the normal and elevated depressive symptom groups. Finally, we explored the impact of anti-depressant treatment by comparing treated versus untreated participants within each group (normal and elevated) using an independent samples t-test or Mann–Whitney U test. Significance was set at p < 0.05. The data are expressed as mean (standard deviation (SD)) unless otherwise specified. Analyses were conducted in SPSS (Version 27).

### Data availability statement

The data are available on reasonable request from the corresponding author.

## Results

### Parkinson’s disease

#### Participants

A total of 194 participants with Parkinson’s were analysed (138 males, median age = 60.66, interquartile range (IQR) = 13.11, range = 38–77 years old).

#### Symptoms of depression

Sixty-three participants (32.5%) scored 5 or more on the PHQ-9 and were assigned to the elevated PHQ-9 group. Symptoms of depression above this threshold were significantly more prevalent in male (37.7%; 52 of 138) than female (19.6%; 11 of 56) participants (p = 0.015).

#### Normal versus elevated depressive symptom groups

There were no significant differences between participants with normal or elevated PHQ-9 scores with regard to their disease duration, levodopa equivalent daily dose or motor severity as measured by the MDS-UPDRS Part 3 OFF or ON dopaminergic medication. Those with elevated PHQ-9 scores were younger (p = 0.022).

The visual analogue scale from the EQ-5D-5L was similar between groups (Table 1). However, participants with an elevated PHQ-9 score self-reported a greater non-motor and motor impact of Parkinson’s on their experiences of daily living compared to those with a normal PHQ-9 score (MDS-UPDRS Part 1b: p < 0.001; MDS-UPDRS Part 2: p = 0.001). They also self-reported a poorer quality of life, as indicated by the PDQ-39 summary index (p < 0.001), individual PDQ-39 dimension scores (Table 1) and EQ-5D-5L index (p < 0.001). An increased burden of non-motor symptoms (NMSS: p < 0.001) was observed in the elevated PHQ-9 group, and this difference remained significant when removing the mood/cognition domain from the NMSS total score (p < 0.001). Motor complications of treatment (MDS-UPDRS Part 4) were more commonly reported by participants with an elevated PHQ-9 score compared to participants with a normal PHQ-9 score (p = 0.049).

**Table 1.** Participants with Parkinson’s were dichotomised into those with normal or elevated PHQ-9 scores and compared against each other using the Mann–Whitney U test.

	Median (IQR) or total (%)			p value
	Entire Cohort (n = 194)	Normal PHQ-9 (n = 131)	Elevated PHQ-9 (n = 63)	
Gender, male (%)	138/194 (71.1)	86/131 (65.6)	52/63 (82.5)	0.015
Age, years	60.7 (13.1)	61.7 (12.3)	58.4 (13.4)	0.022
Disease Duration, years	4.1 (3.7)	3.7 (3.7)	4.2 (3.7)	0.903
Levodopa Equivalent Daily Dose (mg)	475 (393)	480 (375)	475 (500)	0.354
Anti-depressant Use (%)	30/194 (15.5)	17/131 (13.0)	13/63 (20.6)	0.281
*SSRIs*	15/30 (50.0)	8/17 (47.1)	7/13 (53.8)	-
*SNRIs*	4/30 (13.3)	2/17 (11.8)	2/13 (15.4)	-
*NaSSA*	4/30 (13.3)	3/17 (17.6)	1/13 (7.7)	-
*Tricyclic Anti-depressants*	7/30 (23.3)	4/17 (23.5)	3/13 (23.1)	-
Clinician-Reported Outcome Measures				
MDS-UPDRS Part 3 OFF-med	31.0 (18.0)	31.0 (21.0)	31.0 (16.0)	0.592
MDS-UPDRS Part 3 ON-med	18.0 (14.00)	20.0 (14.00)	17.0 (14.00)	0.200
Participant-Reported Outcome Measures				
PHQ-9	3.0 (4.0)	2.0 (3.0)	6.0 (3.0)	<0.001
MDS-UPDRS Part 1B	5.0 (5.0)	4.5 (3.0)	8.0 (6.0)	<0.001
MDS-UPDRS Part 2	6.0 (7.0)	6.0 (6.0)	9.0 (7.0)	0.001
EQ-5D-5L Index	0.89 (0.19)	0.94 (0.17)	0.84 (0.14)	<0.001
EQ-5D-5L Visual Analogue Scale	80.0 (20.0)	80.0 (20.0)	75.0 (15.0)	0.207
PDQ-39				
*Mobility*	2.5 (10.0)	2.5 (7.5)	6.3 (10.6)	0.005
*ADLs*	8.3 (12.50)	4.2 (8.3)	8.3 (20.8)	0.002
*Emotional Well-being*	12.5 (16.7)	8.3 (16.7)	18.8 (25.0)	<0.001
*Stigma*	6.3 (18.8)	6.3 (18.8)	12.5 (31.3)	<0.001
*Social Support*	0.0 (0.0)	0.0 (0.0)	0.0 (16.7)	0.029
*Cognition*	6.3 (18.8)	6.3 (12.5)	18.8 (18.8)	<0.001
*Communication*	0.0 (16.7)	0.0 (10.4)	8.3 (16.7)	0.006
*Bodily Discomfort*	16.7 (33.3)	8.3 (25.0)	25.0 (25.0)	<0.001
*Summary Index*	9.0 (11.7)	6.8 (9.4)	16.41 (13.6)	<0.001
Clinician- and Participant-Reported Outcome Measures^a^				
MDS-UPDRS Part 4	4.0 (6.0)	3.0 (6.0)	5.0 (6.0)	0.049
NMSS	26.0 (33.0)	18.0 (24.0)	36.0 (40.0)	<0.001

IQR: interquartile range; SSRI: selective serotonin reuptake inhibitors; SNRI: serotonin – noradrenaline reuptake inhibitors; NaSSA: noradrenergic and specific serotonergic antidepressants; PHQ-9: Patient Health Questionnaire; MDS-UPDRS: Movement Disorder Society Sponsored Revision of the Unified Parkinson’s Disease Rating Scale; PDQ-39: Parkinson’s Disease Questionnaire-39; NMSS: non-motor symptoms scale.

a Completed by the clinician after a discussion/semi-structured interview with the participant.

When including age, gender, disease duration, motor severity (MDS-UPDRS Part 3) and anti-depressant use into multivariable linear regression models, the PHQ-9 remained the strongest significant predictor of MDS-UPDRS Part 1b (Coefficient: 0.62, 95% confidence interval (CI): 0.46 to 0.77, p < 0.001), Part 2 (Coefficient: 0.50, 95% CI: 0.28 to 0.72, p < 0.001) and Part 4 (Coefficient: 0.24, 95% CI: 0.10 to 0.38, p = 0.001). PHQ-9 scores were significantly predictive of the NMSS minus the mood/cognition domain (Coefficient: 3.77, 95% CI: 2.87 to 4.66, p < 0.001), EQ-5D-5L index (Coefficient: −0.01, 95% CI: −0.02 to −0.01, p < 0.001) and PDQ-39 summary index (Coefficient: 1.46, 95% CI: 1.08 to 1.83, p < 0.001). Table 2 and Supplemental Table S1 present the univariable linear regression models and individual PDQ-39 dimension data, respectively.

**Table 2.** Univariable linear regression models were performed to explore the relationship between depression symptoms and self-reported outcome measures in Parkinson’s. Gender (with male as the reference category), age at baseline, disease duration from diagnosis, anti-depressant use, and motor severity (MDS-UPDRS Part 3) were included in the model as predictors.

	Coefficient (95% CI)	p value
Model 1: MDS-UPDRS Part 1B		
PHQ-9	0.62 (0.47 to 0.76)	<0.001
Gender	–0.91 (–2.05 to 0.24)	0.121
Age	0.00 (–0.05 to 0.06)	0.899
Diagnosis Duration	0.22 (0.04 to 0.40)	0.018
MDS-UPDRS Part 3 ON-med	0.03 (–0.02 to 0.08)	0.222
Anti-depressant Use	1.38 (–0.02 to 2.77)	0.052
Model 2: MDS-UPDRS Part 2		
PHQ-9	0.49 (0.27 to 0.72)	<0.001
Gender^a^	–1.74 (–3.29 to −0.19)	0.028
Age	0.07 (–0.00 to 0.15)	0.056
Diagnosis Duration	0.45 (0.21 to 0.69)	<0.001
MDS-UPDRS Part 3 ON-med	0.13 (0.06 to 0.20)	<0.001
Anti-depressant Use	1.03 (–0.89 to 2.96)	0.291
Model 3: MDS-UPDRS Part 4		
PHQ-9	0.27 (0.12 to 0.42)	0.001
Gender	0.04 (–0.99 to 1.07)	0.936
Age	–0.09 (–0.14 to −0.04)	<0.001
Diagnosis Duration	0.44 (0.29 to 0.59)	<0.001
MDS-UPDRS Part 3 OFF-med	0.07 (0.04 to 0.11)	<0.001
Anti-depressant Use	0.19 (–1.07 to 1.44)	0.771
Model 4: NMSS (Minus Domain 3)^b^		
PHQ-9	3.69 (2.82 to 4.56)	<0.001
Gender^a^	–11.57 (–18.21 to −4.92)	<0.001
Age	0.28 (–0.05 to 0.61)	0.097
Diagnosis Duration	0.89 (–0.19 to 1.97)	0.107
MDS-UPDRS Part 3 ON-med	0.34 (0.04 to 0.64)	0.025
Anti-depressant Use	2.42 (–5.98 to 10.82)	0.571
Model 5: EQ-5D-5L Index		
PHQ-9	–0.01 (–0.02 to 0.01)	<0.001
Gender	0.003 (–0.04 to 0.04)	0.884
Age	0.00 (–0.002 to 0.002)	0.930
Diagnosis Duration	–0.01 (–0.02 to −0.004)	0.001
MDS-UPDRS Part 3 ON-med	–0.002 (–0.004 to 0.00)	0.020
Anti-depressant Use	–0.07 (–0.12 to −0.02)	0.004
Model 6: PDQ-39 Summary Index		
PHQ-9	1.50 (1.14 to 1.86)	<0.001
Gender	–0.86 (–3.60 to 1.89)	0.539
Age	–0.11 (–0.25 to 0.03)	0.113
Diagnosis Duration	0.39 (–0.05 to 0.83)	0.082
MDS-UPDRS Part 3 ON-med	0.04 (–0.08 to 0.17)	0.514
Anti-depressant Use	2.71 (–0.68 to 6.10)	0.116

CI: confidence interval; PHQ-9: Patient Health Questionnaire; MDS-UPDRS: Movement Disorder Society Sponsored Revision of the Unified Parkinson’s Disease Rating Scale; PDQ-39: Parkinson’s Disease Questionnaire-39; NMSS: non-motor symptoms scale.

a Male participants self-reported higher scores on these assessments compared to female participants.

b Domain 3 measures mood/cognition and was therefore omitted as it would strongly correlate with the PHQ-9.

#### Impact of anti-depressant use

At baseline, ongoing treatment with anti-depressants was observed in 13 of 63 (20.6%) participants with an elevated PHQ-9 score, and in 17 of 131 participants (13.0%) with a normal PHQ-9 score (Supplemental Table S2). Compared to treated participants with a normal PHQ-9 score, treated participants with an elevated PHQ-9 score self-reported a greater impact of Parkinson’s on their non-motor experiences of daily living (MDS-UPDRS Part 1b: p = 0.010), an increased burden of non-motor symptoms (NMSS: p = 0.008) and a poorer quality of life (PDQ-39 summary index: p = 0.008; EQ-5D-5L visual analogue scale: p = 0.054). There were no significant differences in how the anti-depressant treated participants with normal or elevated depressive symptoms completed the EQ-5D-5L index or MDS-UPDRS Parts 2 and 4. The MDS-UPDRS Part 3 OFF-medication did not differ between treated and untreated participants, regardless of depression status, p = 0.690.

### Multiple system atrophy

#### Participants

Fifty participants with multiple system atrophy took part (24 males, mean age = 63.30 years, SD = 7.86, range = 49–78 years old). Twenty-eight (56.0%) participants were classified as having the parkinsonian-predominant phenotype and 22 (44.0%) were classified as having the cerebellar-predominant phenotype.

#### Symptoms of depression

Twenty-one participants (42.0%) scored 14 or above on the BDI-II and were assigned to the elevated BDI-II group. Symptoms of depression above this threshold occurred at a similar frequency in participants with the parkinsonian phenotype (42.9%; 12 of 28) or cerebellar phenotype (40.9 %; 9 of 22), p = 0.890. Symptoms of depression were significantly more prevalent in female (61.5%; 16 of 26) than male (20.8%; 5 of 24) participants (p = 0.004).

#### Normal versus elevated depressive symptom groups

There were no significant differences between participants with a normal or elevated BDI-II score with regard to their age at baseline, disease duration from diagnosis, and overall disease severity as measured by the UMSARS (Table 3). Participants with an elevated BDI-II score did, however, self-report worse health-related quality of life compared to those with a normal BDI-II score. The motor (p = 0.002), non-motor (p = 0.008) and emotional/social functioning domains of the MSA-QoL (p < 0.001) were significantly different between these groups, in addition to the visual analogue scale (p = 0.009).

**Table 3.** Participants with multiple system atrophy were dichotomised into those with normal or elevated BDI-II scores and compared against each other. The mean (SD) is given unless stated otherwise.

	Mean (SD) or total (%)			p value
	Entire Cohort (n = 50)	Normal BDI-II (n = 29)	Elevated BDI-II (n = 21)	
Gender, male (%)	24/50 (48.0)	19/29 (65.5)	5/21 (23.8)	0.004
Age, years	63.3 (7.9)	62.8 (8.4)	64.0 (7.2)	0.611
Disease Duration, years^a^	0.8 (1.1)	0.9 (1.5)	0.7 (0.9)	0.180
Levodopa Equivalent Daily Dose (mg)	626 (372)	597 (443)	664 (263)	0.634
Anti-depressant Use (%)	18/50 (36.0)	10/29 (34.5)	8/21 (38.1)	0.793
*SSRIs*	10/18 (55.6)	5/10 (50.0)	5/8 (62.5)	-
*SNRIs*	1/18 (5.6)	1/10 (10.0)	0/8	-
*NaSSA*	4/18 (22.2)	2/10 (20.0)	2/8 (25.0)	-
*Tricyclic Anti-depressants*	3/18 (16.7)	2/10 (20.0)	1/8 (12.5)	-
Clinician Reported Outcome Measures				
UMSARS Part 1^b^	20.6 (5.4)	20.7 (5.7)	20.6 (5.1)	0.958
UMSARS Part 2	23.8 (5.51)	24.3 (6.2)	23.0 (4.4)	0.412
UMSARS Total	44.9 (9.8)	45.0 (10.8)	43.6 (8.5)	0.626
Participant-Reported Outcome Measures				
BDI-II^a^	12.0 (11.0)	8.0 (5.0)	18.0 (6.0)	<0.001
MSA-QoL (Motor)	38.3 (17.4)	32.0 (16.7)	46.9 (14.8)	0.002
MSA-QoL (Non-motor)	37.2 (16.2)	32.1 (14.2)	44.2 (16.6)	0.008
MSA-QoL (Emotion/Social Functioning)^a^	29.5 (29.9)	21.4 (20.5)	44.6 (38.4)	<0.001
MSA-QoL Visual Analogue Scale	51.3 (23.0)	58.4 (19.7)	41.4 (24.0)	0.009

SD: standard deviation; SSRI: selective serotonin reuptake inhibitors; SNRI: serotonin – noradrenaline reuptake inhibitors; NaSSA: noradrenergic and specific serotonergic antidepressants; BDI-II: Beck Depression Inventory-II; UMSARS: unified multiple system atrophy rating scale; MSA-QoL: multiple system atrophy quality of life scale.

a Median (IQR).

b Completed by the clinician after a discussion with the participant and/or carer.

The univariable linear regression analyses are presented in Table 4. In a multivariable linear regression model, the BDI-II remained a strong significant predictor of health-related quality of life even after adjustment for age, gender, disease duration, objective motor severity (UMSARS Part 2) and anti-depressant use (Motor Domain: coefficient 1.28, 95% CI: 0.67 to 1.90, p < 0.001; Non-motor Domain: coefficient: 0.90, 95% CI: 0.26 to 1.53, p = 0.007; Emotional/Social Functioning Domain: coefficient: 2.30, 95% CI: 1.67 to 2.93, p < 0.001; visual analogue scale: coefficient: –1.19, 95% CI: –1.96 to −0.41, p = 0.002).

**Table 4.** Univariable linear regression models were performed to explore predictors of health-related quality of life in multiple system atrophy. Gender (with male as the reference category), age, diagnosis duration, objective motor severity (UMSARS Part 2), anti-depressant use and BDI-II scores were separately entered into the model for each sub-scale of the MSA-QoL. Please note that higher scores on the MSA-QoL reflect poorer quality of life, while a higher score on the visual analogue scale indicates a higher level of life satisfaction.

	Beta coefficient (95% CI)	p value
Model 1: MSA-QoL (Motor)		
BDI-II	1.17 (0.54 to 1.80)	0.001
Gender	3.02 (–6.96 to 13.00)	0.546
Age	–0.10 (–0.74 to 0.55)	0.762
Diagnosis Duration	–1.60 (–7.44 to 4.25)	0.585
UMSARS Part 2	1.04 (0.17 to 1.91)	0.020
Anti-depressant Use	1.93 (8.49 to 12.34)	0.711
Model 2: MSA-QoL (Non-motor)		
BDI-II	0.97 (0.37 to 1.58)	0.002
Gender	7.28 (–1.80 to 16.35)	0.114
Age	0.01 (–0.59 to 0.61)	0.966
Diagnosis Duration	–2.65 (–8.05 to 2.75)	0.328
UMSARS Part 2	0.34 (–0.51 to 1.19)	0.425
Anti-depressant Use	4.53 (–5.08 to 14.13)	0.348
Model 3: MSA-QoL (Emotion/Social Functioning)		
BDI-II	2.39 (1.75 to 3.02)	<0.001
Gender	13.66 (1.21 to 26.11)	0.032
Age	–0.14 (–0.98 to 0.70)	0.744
Diagnosis Duration	–5.35 (–12.84 to 2.14)	0.157
UMSARS Part 2	0.32 (–0.88 to 1.51)	0.594
Anti-depressant Use	3.20 (–10.37 to 16.77)	0.638
Model 4: MSA-QoL (Visual Analogue Scale)		
BDI-II	–1.44 (–2.29 to −0.59)	0.001
Gender	–15.49 (–27.94 to −3.04)	0.016
Age	–0.27 (–1.12 to 0.57)	0.521
Diagnosis Duration	3.38 (–4.30 to 11.06)	0.380
UMSARS Part 2	–0.37 (–1.58 to 0.84)	0.539
Anti-depressant Use	–17.89 (–2.82 to −5.13)	0.007

CI: confidence interval; BDI-II: Beck Depression Inventory-II; UMSARS: unified multiple system atrophy rating scale; MSA-QoL: multiple system atrophy quality of life scale.

#### Impact of anti-depressant use

Ongoing treatment with anti-depressants was observed in 8 of 21 (38.1%) participants with an elevated BDI-II score, and in 10 of 29 participants (34.5%) with a normal BDI-II score (Supplemental Table S2). For the MSA-QoL motor domain and UMSARS Part 1, no significant differences were observed between the normal and elevated BDI-II scoring participants receiving anti-depressant treatment (p > 0.05). Compared to treated participants with a normal BDI-II score, treated participants with an elevated BDI-II score self-reported that the non-motor (p = 0.023) and emotion/social functioning aspects of life were significantly worse (p = 0.014). The treated participants with an elevated BDI-II score were also less satisfied with life as indicated by a lower visual analogue score (p = 0.001). The UMSARS Part 2 scores were not significantly different between the treated versus untreated participants, regardless of depression status (p = 0.685).

## Discussion

Participant-reported outcome measures are routinely implemented as clinical trial endpoints. Not only are they time- and cost-effective, but they also enrich our understanding of the participants’ experience with information that cannot be gained from biomedical tests or clinician-rated assessments alone.^19^ Depression may be a psychological reaction to a diagnosis or to the presence of motor/non-motor disability, or may be a manifestation of the neurodegenerative process itself. The converse is also true in that mood states can influence how people perceive and process information^20^ and this must be fully considered in the evaluation of trial outcomes.

People with depression demonstrate mood congruent cognitive biases, characterised by diminished attention to positive stimuli and difficulty disengaging attention from negative stimuli.^21,22^ They interpret ambiguous information more pessimistically and have a memory bias for negative information.^23,24^ Participants with an elevated depression score may therefore endorse more negative symptoms on self-reported outcome measures of disease severity and quality of life, even in the absence of severe disability on objective assessments. The complex relationship between mood, functional disability and quality of life should be considered in the outcome measurement of an intervention.

This appears to be the case in our two groups of participants with neurodegenerative diseases who self-reported mild-to-moderate depressive symptoms at baseline. Of fundamental importance is the way in which participant-reported outcomes are captured. The MDS-UPDRS contains clear instructions regarding how each of its subsections are completed and by whom. Parts 1b and 2 are designed to be completed by the participant and/or their caregiver (with the caveat of being reviewed by the clinician for completeness and clarity) and are therefore vulnerable to more pessimistic reporting because of depressed mood, especially if completed by the participant alone. The fact that depressive symptoms significantly predicted poorer outcomes on the MDS-UPDRS Parts 1b and 2, even after adjusting for objective motor severity and disease duration, supports this hypothesis. By contrast, UMSARS Part 1 scores were decided by the clinician ‘according to a patient and caregiver interview’ and were consequently not influenced by depression status. However, the elevated PHQ-9 group in the Parkinson’s cohort self-reported a greater burden of non-motor symptoms, despite the scores being obtained through clinician-led interview. It could be argued that the participant is, to some extent, still guiding such scoring. While the clinician may ask if they have experienced any given non-motor symptom, they would not decide its frequency or severity on behalf of the participant. The questionnaire is therefore conducted in a different manner to UMSARS Part 1, with the latter relying more on the clinician indicating a score that best fits the participant’s condition after an open-ended discussion.

There may of course be other factors which influence participant-reported outcomes. Certain response styles can significantly impact on the data collected whereby participants systematically select options that may not reflect their true opinions (e.g. tendency to always choose the middle response category or selecting the most extreme options).^25^ The cultural or socioeconomic backgrounds of participants could also influence responses or understanding of questions, although this explanation is less likely given that the majority of our sample were White British with more than 12 years of education.

Certainly, it could just be that people with depression actually have more advanced disease progression. This is unlikely to be the sole explanation for our findings given that clinician-rated motor severity, disease duration and total levodopa equivalent daily dose was comparable between participants with a normal or elevated depression score, suggesting that negative self-reporting was not entirely a reactive response to severe motor impairment. While the MDS-UPDRS and UMSARS were unsurprisingly associated with several participant-reported questionnaires, depression scores were a much stronger predictor of these outcomes overall.

We readily acknowledge, however, that collinearity may exist between all of the variables studied here. It raises the possibility of a bi-modal effect or dichotomy of ‘what came first’, and it could be that the presence of other non-motor symptoms or a poorer quality of life are the driving factors for developing depression. Nonetheless, previous research in Parkinson’s has demonstrated that depression itself is the most significant predictor of quality of life variability, and that the impact of depression exceeds that of motor severity.^26,27^ Presurgical depression has also been associated with a negative self-reported outcome of deep brain stimulation therapy for Parkinson’s, despite significant motor improvements.^28^ A randomised controlled trial of paroxetine, nortriptyline and placebo for treatment of Parkinson’s depression corroborates these findings. Patients who experienced improvements in depression, compared to those who did not, also experienced significant improvements in measures of quality of life and disability.^29^ Thus, the evolution of neuropsychiatric symptoms does not appear to follow the progression of motor problems (or autonomic dysfunction in multiple system atrophy). While understanding the aetiology of depression in neurodegenerative diseases goes beyond the scope of the current work, depression may be an integral part of the clinical spectrum rather than a symptom secondary to functional disability.^8^ Our own findings appear to align with these conclusions, highlighting that depressive symptoms are a major influence on self-perceived disease severity and quality of life.

These findings may have substantial implications when interpreting participant-reported outcome measures as primary endpoints in clinical trials, and may need to be taken into account when formulating statistical analysis plans.^30^ To avoid a potential bias, while completely capturing the severity of someone’s disease, it may be necessary to objectively capture depression severity at the same time as other participant-reported outcome measures to allow for consideration of statistical adjustments for mood status, in addition to routine confounders such as age or disease duration. It is also important that during the set-up and conduct of trials, raters are adequately trained in the administration of clinical scales (including self-reporting) to ensure there are consistent practices longitudinally throughout trial conduct. Furthermore, the impact of concomitant medications such as anti-depressants administered during trial follow-up needs to be carefully considered. Although we did not find a clear impact of anti-depressant use on self-reporting, it is important to consider that perhaps these participants were not receiving adequate treatment to manage their mood disturbances.

### Limitations and future directions

Depression status was not assessed using structured neuropsychiatric interviews but by the self-reported BDI-II or PHQ-9, which are subjective in nature. An elevated depression score might stem from the physical or non-motor symptoms associated with neurodegenerative diseases (e.g. sleeping difficulties, appetite changes, loss of energy or fatigue) and not necessarily the emotional component behind these symptoms such as abulia or anhedonia.^5^ It is therefore possible that some participants who scored highly on the depression scale might have answered the questions based primarily on the presence of non-motor symptoms, and what we captured was actually a sub-group with non-levodopa or non-anti-depressant responsiveness. Future investigations are encouraged to perform a detailed exploration of which items contributed to an elevated score. The results should also be interpreted with caution, especially as the rate of clinically diagnosed depression may be lower than the rate found using the BDI-II or PHQ-9 in these cohorts.^5^

It is important to consider if the self-reported data was captured when the participant was OFF or ON dopaminergic medication. Mood disturbances have been associated with motor fluctuations, and perhaps reflect a psychological reaction to motor dysfunction or a manifestation of changing dopamine levels. While Parkinson’s participants completed these questionnaires during the transition period or when ON-medication, we cannot exclude that their responses were not influenced by some residual effects from being OFF-medication.

## Conclusion

In clinical trials for neurodegenerative diseases, we have shown that participants with mild-to-moderate depressive symptoms self-report that their quality of life and disability is more severely affected than participants scoring normally on validated mood scales. While this may be explicable in terms of a non-motor manifestation of their disease or a greater emotional reaction to the consequences of their illness, there is a clear impact on the affected individuals that is not fully captured by a clinician’s objective scoring of motor severity. This needs to be carefully considered when planning the design and analysis of trials with respect to both emphasising participant-reported outcomes and adjusting clinician-rated outcomes according to the concurrent level of depression. In parallel with the conduct of trials, the detection of mild-to-moderate depression should also trigger a discussion regarding the role of pharmacological and non-pharmacological interventions to help relieve mood disturbances.

## Supplemental Material

sj-docx-1-ctj-10.1177_17407745251387571 – Supplemental material for Mild-to-moderate depressive symptoms impact on self-reported outcome measures in clinical trials for neurodegenerative diseases Supplemental material, sj-docx-1-ctj-10.1177_17407745251387571 for Mild-to-moderate depressive symptoms impact on self-reported outcome measures in clinical trials for neurodegenerative diseases by Christine Girges, Nirosen Vijiaratnam, Alexa King, Grace Auld, Rachel McComish, Kashfia Chowdhury, Gareth Ambler, Kate Maclagan, Patricia Limousin, Dilan Athauda, Huw R Morris, Vincenzo Libri and Thomas Foltynie in Clinical Trials
