# Supplementary material for: Mild-to-moderate depressive symptoms impact on self-reported outcome measures in clinical trials for neurodegenerative diseases
Source: Clin Trials. 2025 Nov 30;23(1):54–64. doi: 10.1177/17407745251387571 (PMC12909607; doi:10.1177/17407745251387571)
Supplement: sj-docx-1-ctj-10.1177_17407745251387571 – Supplemental material for Mild-to-moderate depressive symptoms impact on self-reported outcome measures in clinical trials for neurodegenerative diseases [file sj-docx-1-ctj-10.1177_17407745251387571.docx]

**SUPPLEMENTARY INFORMATION**

**Supplementary Table 1.** Univariable linear regression models were performed to explore the relationship between depression symptoms and Parkinson’s Disease Questionnaire-39 (PDQ-39) dimension scores in Parkinson’s. Gender (with male as the reference category), age, disease duration from diagnosis, anti-depressant use, and motor severity (MDS-UPDRS Part 3) were included in the model as predictors.

| **Model 1: PDQ-39 Mobility** | **Coefficient (95% CI)** | **P-value** |
| --- | --- | --- |
| PHQ-9 | 1.04 (0.54 to 1.55) | <0.001 |
| Gender | 2.26 (-1.16 to 5.68) | 0.195 |
| Age | -0.12 (-0.29 to 0.05) | 0.155 |
| Diagnosis Duration | 0.50 (-0.04 to 1.04) | 0.071 |
| MDS-UPDRS Part 3 ON-med | 0.01 (-0.14 to 0.15) | 0.929 |
| Anti-depressant Use | 2.90 (-1.32 to 7.12) | 0.177 |
| **Model 2: PDQ-39 ADLs** |  |  |
| PHQ-9 | 1.19 (0.70 to 1.68) | <0.001 |
| Gender | -3.11 (-6.49 to 0.27) | 0.071 |
| Age | 0.08 (-0.09 to 0.25) | 0.351 |
| Diagnosis Duration | 0.39 (-0.15 to 0.93) | 0.152 |
| MDS-UPDRS Part 3 ON-med | 0.20 (0.05 to 0.35) | 0.011 |
| Anti-depressant Use | 1.38 (-2.78 to 5.54) | 0.513 |
| **Model 3: PDQ-39 Emotional Well-being** |  |  |
| PHQ-9 | 2.09 (1.50 to 2.68) | <0.001 |
| Gender | 0.82 (-3.52 to 5.15) | 0.711 |
| Age | -0.24 (-0.45 to -0.03) | 0.028 |
| Diagnosis Duration | 0.05 (-0.64 to 0.74) | 0.886 |
| MDS-UPDRS Part 3 ON-med | -0.09 (-0.29 to 0.10) | 0.348 |
| Anti-depressant Use | 6.85 (1.65 to 12.05) | 0.010 |
| **Model 4: PDQ-39 Stigma** |  |  |
| PHQ-9 | 1.66 (0.86 to 2.46) | <0.001 |
| Gender | -0.93 (-6.41 to 4.54) | 0.737 |
| Age | -0.60 (-0.86 to -0.35) | <0.001 |
| Diagnosis Duration | 0.15 (-0.72 to 1.02) | 0.738 |
| MDS-UPDRS Part 3 ON-med | -0.02 (-0.27 to 0.22) | 0.852 |
| Anti-depressant Use | -3.07 (-9.74 to 3.60) | 0.365 |
| **Model 5: PDQ-39 Social Support** |  |  |
| PHQ-9 | 0.73 (0.32 to 1.15) | 0.001 |
| Gender | -0.15 (-2.94 to 2.65) | 0.918 |
| Age | -0.15 (-0.29 to -0.01) | 0.031 |
| Diagnosis Duration | 0.05 (-0.40 to 0.49) | 0.838 |
| MDS-UPDRS Part 3 ON-med | -0.05 (-0.18 to 0.08) | 0.434 |
| Anti-depressant Use | 1.48 (-1.93 to 4.89) | 0.392 |
| **Model 6: PDQ-39 Cognition** |  |  |
| PHQ-9 | 2.10 (1.58 to 2.62) | <0.001 |
| Gender | -2.57 (-6.50 to 1.35) | 0.197 |
| Age | 0.11 (-0.09 to 0.30) | 0.292 |
| Diagnosis Duration | 0.47 (-0.16 to 1.10) | 0.145 |
| MDS-UPDRS Part 3 ON-med | 0.02 (-0.16 to 0.20) | 0.820 |
| Anti-depressant Use | 3.91 (-0.87 to 8.68) | 0.108 |
| **Model 7: PDQ-39 Communication** |  |  |
| PHQ-9 | 1.40 (0.82 to 1.96) | <0.001 |
| Gender* | -5.36 (-9.26 to -1.45) | 0.007 |
| Age | -0.04 (-0.24 to 0.16) | 0.682 |
| Diagnosis Duration | 0.54 (-0.08 to 1.16) | 0.089 |
| MDS-UPDRS Part 3 ON-med | 0.04 (-0.14 to 0.22) | 0.638 |
| Anti-depressant Use | 0.19 (-4.70 to 5.08) | 0.939 |
| **Model 8: PDQ-39 Bodily Discomfort** |  |  |
| PHQ-9 | 2.08 (1.22 to 2.93) | <0.001 |
| Gender | -0.37 (-6.32 to 5.58) | 0.903 |
| Age | 0.22 (-0.08 to 0.51) | 0.148 |
| Diagnosis Duration | 0.90 (-0.04 to 1.83) | 0.061 |
| MDS-UPDRS Part 3 ON-med | 0.17 (-0.10 to 0.43) | 0.213 |
| Anti-depressant Use | 5.70 (-1.52 to 12.92) | 0.121 |

*Male participants self-reported higher scores on this assessment compared to female participants.

**Supplementary Table 2.** Comparison of anti-depressant treated participants with a normal or elevated depression score according to the PHQ-9 for Parkinson’s and BDI-II for multiple system atrophy.

| **Outcome Measure** | **Median (IQR) or total (%)** | | ***p* value** |
| --- | --- | --- | --- |
|  | **Normal Depression Score** | **Elevated Depression Score** |  |
| Disease Population | Parkinson’s disease | Parkinson’s disease | - |
| *N* (%) | 17/131 (13.0) | 13/63 (20.6) | - |
| MDS-UPDRS Part 1b | 5.0 (4.0) | 9.0 (5.0) | 0.010 |
| MDS-UPDRS Part 2 | 6.0 (7.0) | 10.0 (7.0) | 0.365 |
| MDS-UPDRS Part 3 OFF-med | 37.0 (22.0) | 30.0 (18.0) | 0.586 |
| MDS-UPDRS Part 3 ON-med | 20.0 (19.0) | 14.0 (11.0) | 0.232 |
| MDS-UPDRS Part 4 | 3.0 (6.0) | 5.0 (6.0) | 0.291 |
| NMSS | 27.0 (27.0) | 45.0 (55.0) | 0.008 |
| EQ-5D-5L Index | 0.8 (0.2) | 0.8 (0.2) | 0.077 |
| EQ-5D-5L Visual Analogue Scale | 75.0 (15.0) | 70.0 (25.0) | 0.054 |
| PDQ-39 Summary Index | 10.4 (14.1) | 27.7 (10.8) | 0.008 |
|  | **Mean (SD) or total (%)** | |  |
| Disease Population | Multiple system atrophy | Multiple system atrophy | - |
| *N* (%) | 10/29 (34.5) | 8/21 (38.1) | - |
| UMSARS Part 1 | 21.4 (6.0) | 18.0 (4.4) | 0.199 |
| UMSARS Part 2 | 25.9 (5.9) | 20.1 (3.2) | 0.025 |
| MSA-QoL (Motor) | 36.4 (18.4) | 43.3 (16.7) | 0.424 |
| MSA-QoL (Nonmotor) | 32.2 (15.1) | 50.0 (15.0) | 0.023 |
| MSA-QoL (Emotion/Social Functioning) * | 18.8 (25.5) | 49.1 (46.0) | 0.014 |
| MSA-QoL Visual Analog Scale | 53.4 (14.9) | 22.9 (14.7) | 0.001 |

*Median (IQR)

Abbreviations: MDS-UPDRS = Movement Disorder Society Sponsored Revision of the Unified Parkinson’s Disease Rating Scale, PDQ-39 = Parkinson’s Disease Questionnaire-39; NMSS = Non-motor Symptoms Scale, UMSARS = Unified Multiple System Atrophy Rating Scale, MSA-QoL = Multiple System Atrophy Quality of Life Scale.
